# Authors’ Reply

**DOI:** 10.18502/jovr.v16i3.9451

**Published:** 2021-07-29

**Authors:** Kaveh Abri Aghdam, Amin Zand, Mostafa Soltan Sanjari

**Affiliations:** Eye Research Center, The Five Senses Institute, Rassoul Akram Hospital, Iran University of Medical Sciences, Tehran, Iran

1. Dear Editor,

We sincerely thank Dr Guzzi for the interest in our case report^[1]^ and would like to take this opportunity to make some clarifications as follows:

The main concern raised by Dr Guzzi was that serum lead level alone does not adequately reflect the total body burden of the lead and the whole-blood lead level in conjugation with its urinary levels is a primary measure of lead exposure in humans.^[2,3,4]^ We fully agree with Dr Guzzi based on the available literature on this subject. However, one month before the referral, our patient had been diagnosed with lead poisoning just based on serum lead level and had undergone chelation therapy in another tertiary center by a neurologist. Shortly after the completion of treatment, he came to our neuro–ophthalmology clinic for the evaluation of persistent blurred vision in both eyes. Therefore, we could only rely on his previous clinical and paraclinical documents.

In the letter written by Dr Guzzi, the term “papilledema" was used for the description of our patient's ocular condition. However, as we mentioned in our article, papilledema is defined as optic disc edema due to increased intracranial pressure (ICP) and should be differentiated from papillitis. The lumbar puncture in this patient showed that ICP was within normal limits, which ruled out papilledema. Therefore, we considered the condition of the patient as bilateral hemorrhagic optic disc swelling.^[1]^

This is an open access journal, and articles are distributed under the terms of the Creative Commons Attribution-NonCommercial-ShareAlike 4.0 License, which allows others to remix, tweak, and build upon the work non-commercially, as long as appropriate credit is given and the new creations are licensed under the identical terms.

## References

[B1] Abri Aghdam K, Zand A, Soltan Sanjari M (2019). Bilateral optic disc edema in a patient with lead poisoning. J Ophthalmic Vis Res.

[B2] Guzzi G, Spadari F, Bombeccari GP, Pigatto PD (2017). Maxillofacial gunshot wounds and diagnostic tests for lead in the blood. Br J Oral Maxillofac Surg.

[B3] Pigatto PD, Ronchi A, Guzzi G (2016). Iron overload, G6PD deficiency, and lead levels on blood smears. Int J Hematol.

[B4] Casarett LJ, Doull J, Klaassen CD, 6th edition New York: McGraw-Hill Medical Pub.

